# Gas Chromatographic-Ion Mobility Spectrometry Combined with Chemometrics to Study the Changes in Characteristic Odor Components of Galli gigerii Endothelium Corneum in Different Processing Methods

**DOI:** 10.1155/2023/2259280

**Published:** 2023-08-07

**Authors:** Yongqi Zhao, Zhenling Zhang, Hongwei Zhang, Yanbang Shi, Yiming Wang

**Affiliations:** ^1^College of Pharmacy, Henan University of Chinese Medicine, Zhengzhou 450046, China; ^2^Henan Integrated Engineering Technology Research Center of Traditional Chinese Medicine Production, Zhengzhou 450046, China

## Abstract

Galli gigerii endothelium corneum (GGEC) is a traditional Chinese medicine commonly used in clinical practice to treat various conditions such as indigestion, vomiting, spermatorrhea, and enuresis. In this study, the volatile components of different concoctions of GGEC were examined by gas chromatography-ion mobility spectrometry (GC-IMS), and the changes of the components were compared by fingerprinting, combined with principal component analysis (PCA) and orthogonal partial least squares-discriminant analysis (OPLS-DA) to analyze the main volatile components and find out the different markers that can distinguish the different concoctions of GGEC. In the result, the GC-IMS fingerprints of GGEC and its different concoctions showed differences in their volatile components, of which 49 volatiles were clearly characterized, with some components including monomers and dimers. The characteristic volatile components of raw GGEC (SP) were n-nonanal, (E)-2-octenal, beta-ocimene, 2-ethyl-1-hexanol, etc. The characteristic volatile components of stir-fried GGEC (QC) are heptanal, 2-octanol, (E)-2-heptenal, etc. The characteristic volatile components of sand ironing GGEC (ST) are isoamyl acetate, decanal, cyclohexanone, 2-ethyl pyrazine, etc. The characteristic volatile components of stir-fried GGEC with vinegar (CZ) are thiazole, linalool, 2,3,5-trimethylpyrazine, etc. The characteristic volatile components of stir-fried GGEC with milk (FH) are 2-methylbutanoic acid, ethyl acetate, ethyl 2-hydroxypropanoate, butyl acetate, etc. By chemometric analysis, components such as n-nonanal, (E)-2-octenal, 2-pentyl-furan, butanal, 1,4-dioxane, and 2-methylpropanoic acid could be used as difference markers to distinguish different concoction products of GGEC. Furthermore, by analyzing different volatile compounds, we can examine the changes in volatile components during processing of GGEC, which can provide experimental data for the identification and establishment of quality standards.

## 1. Introduction

GGEC, or galli gigerii endothelium corneum, refers to the inner wall of the dried sand sac taken from *Gallus gallus domesticus* Brisson. To obtain GGEC, the chicken's gizzard is removed immediately after it is killed. Then, the inner wall is carefully peeled off, washed, and dried [1]. GGEC is a traditional Chinese medicine commonly used in clinical practice to treat various conditions such as indigestion, vomiting, spermatorrhea, and enuresis. Its therapeutic properties have been recognized and utilized for centuries [2]. Its main components include protein, amino acid, polysaccharide, and trace elements[3]. Raw GGEC has a fishy odor, which makes it unsuitable for clinical use without proper processing. Chinese pharmacopoeia recommends two methods of processing GGEC: frying or scalding it in hot sand. In addition, GGEC can be stir-fried with vinegar or milk. These processing methods help to reduce the fishy odor to varying degrees, making it more palatable for consumption [4]. GGEC exhibits an olfactory characteristic commonly described as a “fishy smell,” which can be attributed to its protein, fatty acid, and other constituent components. These components can be easily decomposed, resulting in the production of biological amines, small molecular aldehydes, ketones, alcohols, trimethylamine, ammonia, and other malodorous substances [5]. In the realm of traditional Chinese medicine, various techniques are employed to eliminate odors from animal-based remedies. These methods include heating, adsorption, inclusion, covering, and microbial fermentation. The principle is to cover these foul odors by physical adsorption or by adding aromatic agents or by transforming and breaking down the unpleasant components by chemical reactions. From this point of view, the processing of GGEC using the frying and sand blanching methods aims to eliminate fishy odors by inducing reactions between odor components at high temperatures. Conversely, the processing method with the addition of vinegar and milk involves the addition of complementary ingredients during the heating process to mask the odor.

A diverse range of assays is employed for the purpose of quality control and composition testing of GGEC. The quality of GGEC is primarily regulated by the Chinese pharmacopoeia through the control of alcohol-soluble leachate content. However, this method fails to differentiate between different processed products of GGEC. In addition, the determination of amino acids in GGEC can be achieved through the utilization of high-performance liquid chromatography (HPLC) with precolumn derivatization [6]. Furthermore, the quality of GGEC can be evaluated by analyzing its nucleoside components using UPLC-Q-TOF-MS and HPLC [7]. However, these methods not only necessitate laborious preprocessing of the sample but also fall short in effectively characterizing its olfactory component. Gas chromatography-ion mobility spectrometry (GC-IMS) is a volatile substance detection method that uses different ions with different mobilities under the same conditions to make different ions have different drift time through the electric field, thus achieving sample separation [8]. GC-IMS provides a nonselective response to several common chemical functional groups (alcohols, amines, ketones, and aldehydes) in the positive ion mode, so that most aroma compounds can be detected by it [9]. GC-IMS is a widely used technique in various fields such as food, agriculture, and pharmaceuticals. For example, GC-IMS was employed to identify alterations in the volatile composition of bacon during various processing stages [10]. In addition, it was utilized to investigate the changes in the volatile composition of candied kumquats under different processing methods [11]. In both cases, the expected outcomes were successfully achieved. GGEC exhibits a potent odor, which undergoes significant alterations upon concoction. The primary objective of GGEC concoction is to mitigate its pungent fishy scent. The GC-IMS technique offers a rapid and efficient detection of volatile odor components. It eliminates the need for complex sample extraction procedures, allowing direct testing or crushing of samples for analysis. Therefore, GC-IMS is highly suitable for analyzing the variations in volatile components among different processed products of GGEC.

In this experiment, GC-IMS was utilized to identify and analyze the volatile odor components of SP, QC, ST, CZ, and FH, which are different processed products of GGEC. A fingerprint analysis was conducted to identify the characteristic volatile components of each product, and chemometrics was used to screen for different volatile components. This data-driven approach provided a more objective method for odor discrimination compared to traditional empirical methods. To the best of our knowledge, this is the first report to combine GC-IMS with PCA/OPLS to explore changes in volatile components during the processing of GGEC. This will help in the identification and quality control of its different processed products.

## 2. Materials and Methods

### 2.1. Experimental Instruments

The experimental instruments used were as follows: Flavour Spec® Gas phase ion mobility spectrometer (G.A.S. Co., Germany), BJ-150 type high-speed multifunctional crusher (Zhongxing Weiye Instrument Co., Beijing, China), BSA224S-CW 1/10,000 balance (Sartoris Technology Instrument Co., Ltd., Gottingen Germany), and GZX-9070 type blast drying oven (Bosun Industrial Co., Shanghai, China).

### 2.2. Medicinal Materials

The medicinal materials used were as follows: GGEC (Aisheng Herbs and Tablets Co., Anhui, China, Batch number: 210802); vinegar (Zilin Vinegar Industry Co., Shanxi, China, Batch number: 20210805); and milk (Mengniu Dairy Co., Inner Mongolia, China, Batch number: 20220107).  Table 1 illustrates the preparation of several GGEC processed products.

### 2.3. Experimental Conditions

The sample powder was weighed 1 g (sieved by No. 4) and loaded into a 20 mL headspace injection vial. Headspace injection conditions: incubation at 80°C, 250 r/min for 20 min, injection needle temperature of 85°C, and injection volume of 500 *μ*L. GC detection conditions: column: MXT-5 (0.53 mm × 15 m), column temperature of 60°C, analysis time of 30 min, and carrier gas of high-purity nitrogen (purity ≥99.999%). The carrier gas flow rate program: initial 2.0 mL/min, hold for 2 min, linearly increase to 100.0 mL/min in 2∼20 min, and maintain at 100.0 mL/min in 20∼30 min. IMS conditions: radioactive source of *β*-rays (tritium, 6.5 keV), positive ion mode, drift tube length of 9.8 cm, linear voltage in the tube of 500 V/cm, and drift. The tube temperature was 45°C, the drift gas was high-purity nitrogen (purity ≥99.999%), and the drift gas flow rate was 150 mL/min.

### 2.4. Data Processing

A qualitative analysis of volatile odorants was performed using the instrument's own analysis software, LAV, and the NIST 2014 database and IMS database built into the GC-IMS Library Search software. The Reporter plug-in in LAV was used to directly compare the spectral differences between samples, and the Gallery Plot plug-in was used to compare the fingerprint profiles to visually and quantitatively compare the differences in volatility between samples. On the basis of the spectral analysis, PCA and OPLS-DA were used for the chemometric analysis of the raw data to quantify the changes of volatile components during the preparation of GGEC and to find the markers of differences between the raw and concocted products.

## 3. Results and Discussion

### 3.1. GC-IMS Spectrum Analysis of Different Processed Products of GGEC

**Figure 1:**
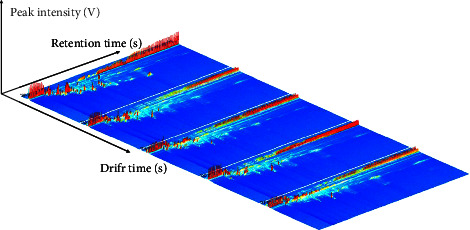
Three-dimensional gas chromatographic ion migration spectrum (GC-IMS) of volatile substances in different processed products of GGEC (SP, CZ, FH, QC, and ST from left to right).

The LAV software's reporter plug-in is utilized to make a direct comparison between samples based on their spectral differences. The data produced by the instrument is three-dimensional, consisting of spectral information on retention time, migration time, and peak intensity (as depicted in Figure 1). The figure shows the distinct differences in volatile components between various processed products of GGEC. However, for the sake of convenience, we have opted to compare these differences from a top-down view.

**Figure 2:**
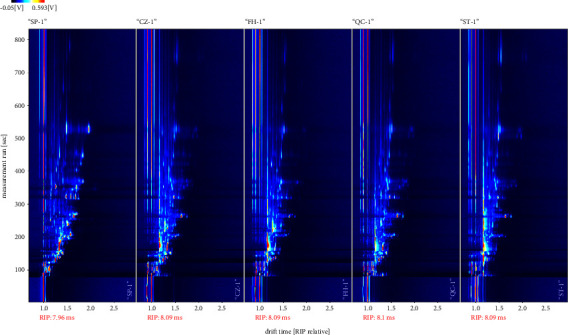
Direct comparison diagram of volatile components in different processed products of GGEC (SP, CZ, FH, QC, and ST from left to right).

In the top viewport (Figure 2), the ordinate represents the retention times of gas chromatography, and the abscissa represents the ion migration time. The background of the whole picture is blue. The red vertical line at abscissa 1.0 is the reactive ion peak (RIP, normalized time point), and each point on both sides of the RIP peak represents a volatile organic compound (VOC). Color represents the concentration of substances, white represents a low concentration, red represents a high concentration, and the darker the color is, the higher the concentration is [12]. It can be seen from the figure that there are significant differences in the volatile components of different processed products of GGEC.

**Figure 3:**
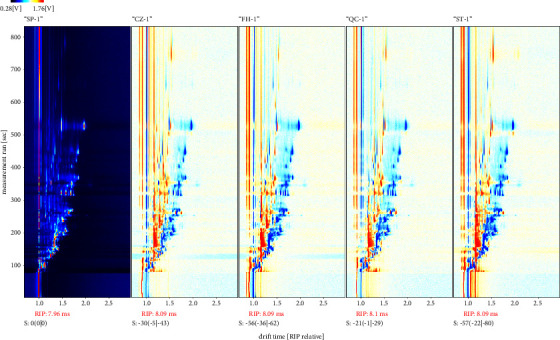
Comparison diagram of volatile matter difference of different processed products of GGEC with raw GGEC as reference deduction (SP, CZ, FH, QC, and ST from left to right).

To facilitate a clearer comparison between various processed products of GGEC, a difference comparison mode was utilized. SP was used as a reference to subtract the spectral information of other samples, allowing for a more intuitive comparison of their differences. If the other samples contain the same VOCs as SP, the background after deduction will be white. If the concentration of a substance in a corresponding area of the sample is above the reference value, the color will be close to red, while if the concentration is below the reference value, the color will be close to blue [13].  Figure 3 illustrates that all samples' spectra contained numerous response signals, with some of them exhibiting significant differences in different GC-IMS spectra. The VOCs of SP and CZ∼ST were notably different, while the VOCs of ST and QC samples were similar.

### 3.2. Qualitative Analysis of VOCs of Different Processed Products of GGEC

According to the gas chromatographic retention time and IMS migration time of the components, the VOCs are qualitatively analyzed in combination with the NIST database and IMS database built in the software [14].  Table 2 lists the qualitative results (55 peaks, for 49 compounds) including compound name, CAS number, molecular formula, molecular weight, RI, retention time, and Dt. Some compounds had two response signals due to the presence of monomers and dimers which showed very similar RI, but the Dt of the two were significantly different due to the various molecular weight. It can be seen that GGEC contains a variety of characteristic volatile components, mainly including alcohol, aldehyde, ketone, acid, and ester. Among them, aldehydes and alcohols account for the majority, followed by esters, ketones, and acids. Six of the 49 compounds exist in GGEC in the form of monomers and dimers at the same time, while the remaining 43 compounds exist in the form of monomers, such as (Z)-3-octen-1-ol, 2-amylfuran, hexyl acetate, and butanoic acid.

### 3.3. GC-IMS Fingerprint Analysis of Different Processed Products of GGEC

**Figure 8:**
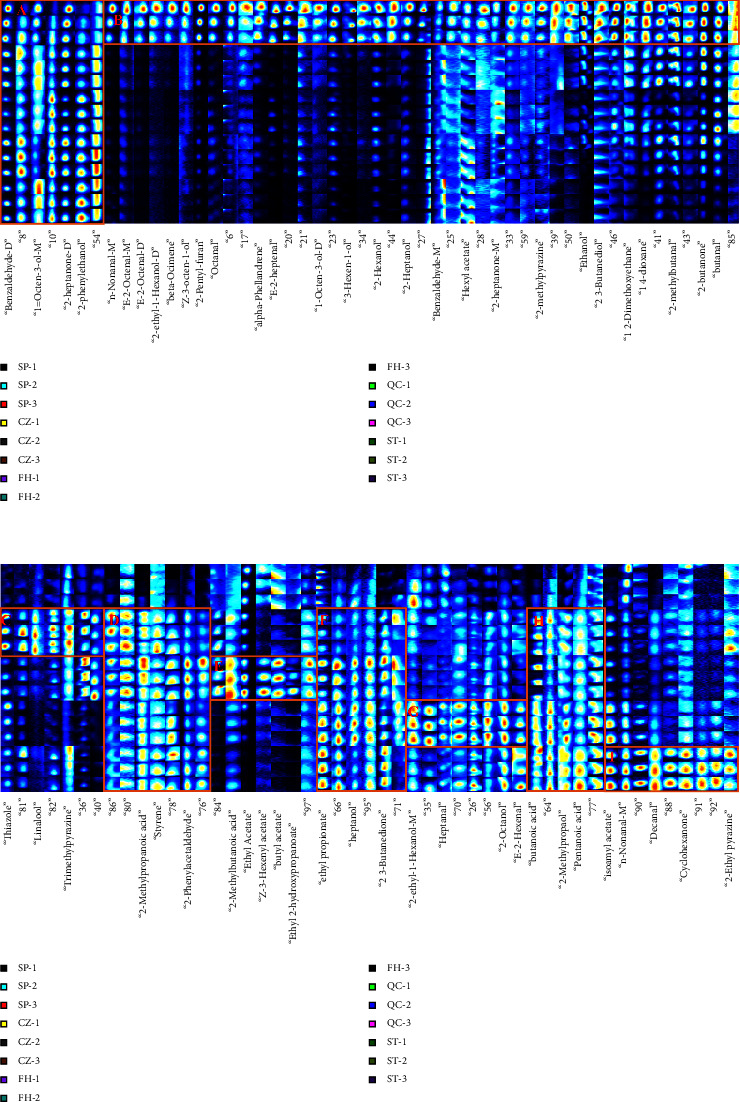
GC-IMS fingerprint of different processed products of GGEC.

To conduct a more comprehensive comparison of the VOCs of various processed products of GGEC, the Gallery Plot plug-in of LAV software was utilized to select five samples, with each sample repeated three times. All peaks to be analyzed in the GC-IMS two-dimensional spectrum will automatically generate a fingerprint, as illustrated in Figure 8. Each row of the fingerprint corresponds to a specific sample, while each column represents a particular VOC, with the content indicated by the color of the corresponding square. At the bottom of each column, we mark the signal peaks with a number (there were 99 signal peaks detected, 55 of which were identified and labeled with component names). Through preliminary comparison of fingerprints, it can be seen that the VOCs of different processed products of GGEC have both common areas and characteristic areas of each sample. Region *A* is the common VOCs area, mainly including benzaldehyde (dimer), 1-octen-3-ol (monomer), 2-heptanone (dimer), 2-phenylethanol, and other components. Region *B* is the characteristic VOCs region of SP, including n-nonanal (monomer), (E)-2-octenal (monomer and dimer), beta-ocimene, 2-ethyl-1-hexanol (dimer), (Z)-3-octen-1-ol, and other components. The disappearance or substantial reduction of these components in other processed products of GGEC indicates that these VOCs have been removed or transformed in the process of processing, and the qualitative VOCs can be considered as identification markers of SP. *D*, *F* , and *H* regions are the common VOCs regions of the four processed products of GGEC, mainly including 2-methylpropanoic acid, styrene, 2-phenylethanol, ethyl propionate, heptanol, 2,3-butanedione, pentanoic acid, and other components. Region *C* is the characteristic VOCs region of CZ, mainly including thiazole, linalool, and 2,3,5-trimethylpyrazine. The *E* region is the characteristic VOCs region of FH, which mainly includes 2-methylbenzoic acid, ethyl acetate, (Z)-3-hexenyl acetate, ethyl 2-hydroxypropanoate, and butyl acetate. *G* region is the characteristic VOCs region of QC, mainly including heptanal, 2-ethyl-1-hexanol (monomer), 2-octanol, and (E)-2-heptenal. Region *I* is the characteristic VOCs region of ST, mainly including isoamyl acetate, n-nonanal (monomer), decanal, cyclohexanone, and 2-ethyl pyrazine.

### 3.4. Chemometric Analysis

To conduct a more in-depth comparison of the differences between various processed products of GGEC and analyze the primary differences in VOCs between samples. SIMCA 14.1 software was utilized to perform principal component analysis (PCA) and orthogonal partial least squares discriminant analysis (OPLS-DA) on the qualitative VOCs. This approach helped identify the characteristic differences among the samples and determine the changes in volatile components of different processed products of GGEC.

#### 3.4.1. Principal Component Analysis (PCA)

**Figure 9:**
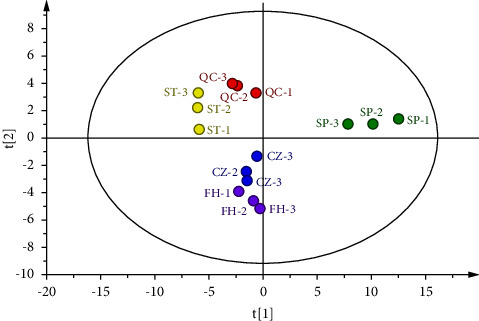
PCA score of different processed products of GGEC.

PCA analysis of VOCs peak areas of different processed products of GGEC was conducted. Five principal components were extracted, with the cumulative interpretation rate of *R*_Xcum_^2^ = 0.971, and the prediction ability of *Q*_cum_^2^ = 0.874, indicating that GGEC and its processed products were well classified. The distance between the circles in Figure 9 reflects the similarity between the corresponding samples [14]. It can be observed that the distribution of each sample shows obvious aggregation and separation. The large gap between SP and other processed products indicates that the VOCs of GGEC have changed greatly after processing. However, the distance between QC and ST and CZ and FH is close, indicating that the difference between them is small.

#### 3.4.2. Orthogonal Partial Least Squares Discriminant Analysis (OPLS-DA)

Based on PCA, OPLS-DA modeling analysis is used to screen the main volatile difference components between GGEC and its different processed products through the value of VIP >1 [15]. SP and QC were analyzed by OPLS-DA, and the three key evaluation indicators of OPLS-DA model quality, *R*_Xcum_^2^ = 0.974, *R*_Ycum_^2^ = 0.992, and *Q*_cum_^2^ = 0.971, showed that the samples were well clustered and significantly separated [9]. Components with a VIP value greater than 1 can significantly affect the classification of SP and QC. The components with a VIP value greater than 1 include 1,4-dioxane, ethanol, 3-hexen-1-ol, n-nonanal (monomer), 2-butanone, 2-methylpropanoic acid, (E)-2-heptanel, 2-pentyl-furan, 2-hexanol, butanoic acid, alpha-phellandrene, (E)-2-octenol (monomer), 2,3-butanedione, butanal, 1,2-dimethoxyethane, 2-methylbutanal, and 2-octanol.

SP and ST were analyzed by OPLS-DA, in which the evaluation index was *R*_Xcum_^2^ = 0.977, *R*_Ycum_^2^ = 0.999, and *Q*_cum_^2^ = 0.996, indicating that the two samples were significantly classified. Among them, the components whose VIP value is predicted to be greater than 1 include 1,4-dioxane, ethanol, 2-butanone, 3-hexen-1-ol, n-nonnal, 2,3-butanedione, 2-hexanol, 2-methylpropanoic acid, (E)-2-heptenal, 2-pentyl-furan, decanal, butanal, 2-methylbutanal, alpha-phellandrene, (E)-2-octenal, 2-phenylethanol, 1,2-dimethoxyethane, butanoic acid, and 2-methylpropanol.

SP and CZ were analyzed by OPLS-DA, in which the evaluation index was *R*_Xcum_^2^ = 0.965, *R*_Ycum_^2^ = 0.991, and *Q*_cum_^2^ = 0.978, indicating that the two samples were significantly classified. Among them, the components whose VIP value is predicted to be greater than 1 include 1,4-dioxane, 3-hexen-1-ol, n-nonanal (dimer), ethanol, 2-methylpropanoic acid, (E)-2-heptenal, 2-hexanol, 2-pentyl-furan, 2,3-butanedione, 2,3,5-trimethylpyrazine, (E)-2-octenal (dimer), 2-butanone, alpha-phellandrene, (E)-2-octenal (monomer), butanal, octanal, n-nonanal (monomer), butanoic acid, and 2-methylpropanol.

The OPLS-DA analysis of SP and FH shows that the evaluation index was *R*_Xcum_^2^ = 0.967, *R*_Ycum_^2^ = 0.999, and *Q*_cum_^2^ = 0.989, indicating that the two samples are significantly classified. The components predicted to be greater than 1 by a VIP value include 1,4-dioxane, ethanol, 2-butanone, 3-hexen-1-ol, n-nonnal (dimer), 2-methylpropanoic acid, 2,3-butanedione, 2-heptanone, 2-pentyl-furan, 2-hexanol, (E)-2-heptenal, alpha-phellandrene, 2-methylbutal, n-nonanal (monomer), (E)-2-octenal (dimer), benzaldehyde, butanal, and (E)-2-octenal (monomer).

### 3.5. Thermographic Analysis

**Figure 10:**
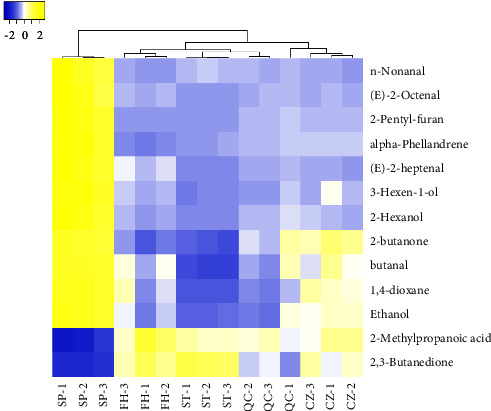
Heat diagram of different processed products of GGEC. Note: The color represents the abundance; from blue to yellow, the abundance value is increasing.

In the analysis of the difference VOCs of different processed products of GGEC, the common difference VOCs are selected for these difference VOCs, which can be used as the difference marker to distinguish SP from other processed products. There are 13 kinds of ingredients in total, including n-nonanal (dimer), (E)-2-octenal (dimer), 2-pentyl-furan, alpha-phellandrene, (E)-2-heptenal, 3-hexen-1-ol, 2-hexanol, 2-butanone, butanal, 1,4-dioxane, ethanol, 2-methylpropanoic acid, and 2,3-butanedione. In order to better compare the variation in composition between samples, the peak area data of the shared differential volatile components of each sample were standardized; that is, the same compound in different samples was standardized using the formula $x_{\text{new}}=\left(x-\bar{x}\right)/\sigma$. Take the standardized results as the chromaticity value, different components as the ordinate, and different processed products of GGEC as the abscissa for thermal map analysis [11]. See Figure 10 for the results. It is obvious from the heat map that the content of 11 components in SP is the highest, including n-nonanal (dimer), (E)-2-octenal (dimer), 2-pentyl-furan, alpha-phellandrene, (E)-2-heptenal, and 1,4-dioxane. However, after processing, the content of these ingredients has decreased to varying degrees. It can be seen that these VOCs have been transformed during the processing process. Among them, n-nonanal has a strong grease gas [16]. (E)-2-Octenal has the aroma of fat and meat [17], 2-pentyl-furan has the smell of beans and earth grass [18], alpha-phellandrene has the smell of peppermint and slight citrus [19], (E)-2-heptenal has the smell of fat [20], 2-hexanol has the strong smell of grass [21], butanal has a suffocating smell, and 1,4-dioxane has the smell of ether [22]. These VOCs together constitute the complex smell of SP.

In processed products, the contents of 2-methylpropanoic acid and 2,3-butanedione have increased. The content of 2-methylpropanoic acid is FH > CZ > ST > QC > SP. The content of 2-methylpropanoic acid in CZ is increased due to the use of vinegar as a processing excipient. However, the content of 2-methylpropanoic acid in ST and QC is also much higher than that in SP, indicating that other substances have been converted into 2-methylpropanoic acid in the process of processing, which is not the only reason for using vinegar as an auxiliary material. The content of 2,3-butanedione is ST > FH > CZ > QC > SP, while the content of ethanol is SP > CZ > QC > FH > ST. Ethanol can be converted to 2,3-butanedione under heated and catalytic conditions, which corresponds to the change in the content of the two components [23]. Vinegar serves a dual purpose, acting not only as a taste-masking agent but also engaging in chemical reactions with specific ingredients. For instance, SP is known to contain a significant quantity of 1,4-dioxane, a chemical compound that has the potential to undergo a reaction with acetic acid under elevated temperatures, resulting in the production of ethylene glycol diacetate [24]. Vinegar, which primarily consists of acetic acid, can be utilized as an additive to enhance the reaction with odor compounds, thereby efficiently eliminating disagreeable odors. Upon analyzing distinct indicators, it was observed that the levels of 2-methylpropanoic acid and 2,3-butanedione experienced a notable rise following GGEC processing. Among the various compounds studied, it was found that short-chain fatty acids, including 2-methylpropanoic acid, exhibited a notable ability to significantly enhance the abundance of fibrolytic bacteria and the corresponding enzymatic activity [25]. By generating butyrate and promoting the growth of beneficial gut bacteria, it is possible to prevent the buildup of lactic acid and maintain intestinal stability [26]. The aforementioned phenomenon has the potential to elucidate the gastrointestinal support properties of GGEC.

## 4. Conclusion

In this paper, GC-IMS was employed to analyze the odor components of different processed products of GGEC. The qualitative identification of VOCs and the analysis of their fingerprints allowed the preliminary assessment of characteristic VOCs in various processed products of GGEC. Subsequently, the differential VOCs in different processed products of GGEC were analyzed using chemometrics. The differential markers were screened by using heat map integration. These markers have the ability to distinguish GGEC from its processed products. In addition, the identification of VOCs can serve as a valuable tool in evaluating the alterations in the odor composition of GGEC during its processing. This information can be utilized to investigate the most effective processing technique for GGEC, with the aim of achieving “deodorization and potency enhancement.”

GC-IMS allows easy, fast, and effective identification of the volatile components of different processed products of GGEC. This analytical technique provides enhanced precision and convenience in distinguishing different GGEC-processed products. As a result, it can be used as a reference for research in the field of GGEC quality control.

## Figures and Tables

**Figure 4:**
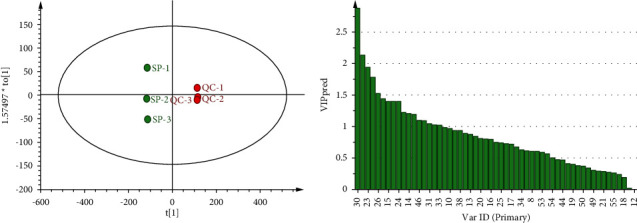
OPLS-DA analysis and VIP score chart of SP and fried QC.

**Figure 5:**
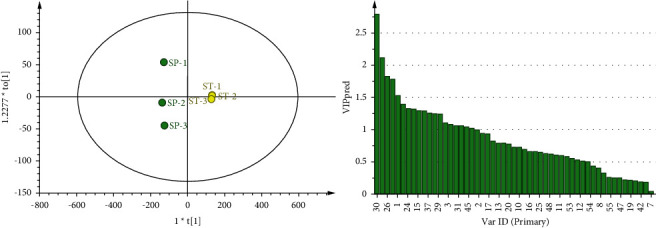
OPLS-DA analysis and VIP score chart of SP and ST.

**Figure 6:**
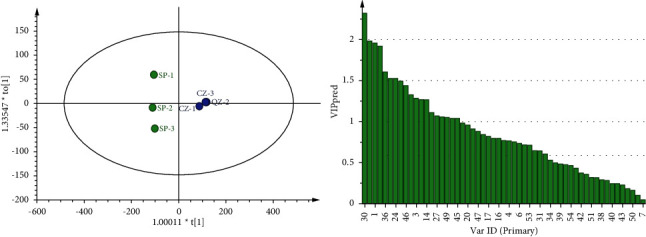
OPLS-DA analysis and VIP score chart of SP and CZ.

**Figure 7:**
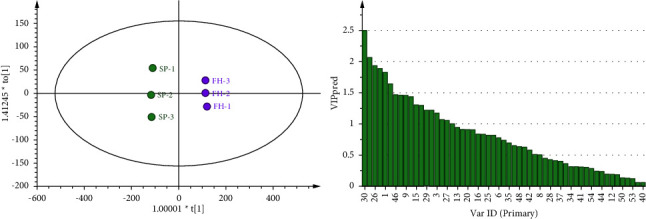
OPLS-DA analysis and VIP score chart of SP and FH.

**Table 1:** Sample preparation methods.

Names	Sample powder	Preparation methods
SP	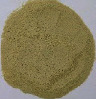	Unprocessed GGEC
QC	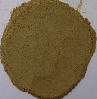	Weigh 100 g of SP, heat the pan over low heat until the temperature is 150°C, add SP, fry until it bulges, remove, and let it cool
ST	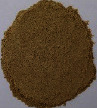	Weigh 100 g of SP and 3 kg of river sand, put the river sand in a pot, stir-fry until the temperature is 250°C, add SP, stir-fry for 80 s and then remove it quickly, sieve the sand, and let it cool
CZ	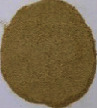	Weigh 100 g of SP and 15 g of vinegar, heat the pot with low heat to 150°C, put SP, fry until it bulges, spray vinegar, take out, and dry
FH	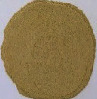	Weigh 100 g of SP and 15 g of milk, heat the pot over moderate heat until the temperature is 150°C, add SP, fry until it bulges, spray milk, remove, and dry

**Table 2:** Qualitative analysis of volatile substances in different processed products of GGEC.

Count	Compounds	CAS	Formulas	MW	RI	Rt (sec)	Dt (RIPrel)	Notes
1	n-Nonanal	C124196	C_9_H_18_O	142.2	1107.1	530.459	1.964	*D*
2	(E)-2-Octenal	C2548870	C_8_H_14_O	126.2	1064.6	456.435	1.343	*M*
3	(E)-2-Octenal	C2548870	C_8_H_14_O	126.2	1061	450.659	1.835	*D*
4	2-Ethyl-1-hexanol	C104767	C_8_H_18_O	130.2	1043.7	423.884	1.797	*D*
5	Beta-ocimene	C13877913	C_10_H_16_	136.2	1030.8	404.985	1.700	
6	(Z)-3-Octen-1-ol	C20125842	C_8_H_16_O	128.2	1046.8	428.610	1.307	
7	2-Ethyl-1-hexanol	C104767	C_8_H_18_O	130.2	1045.8	427.034	1.426	*M*
8	Hexyl acetate	C142927	C_8_H_16_O_2_	144.2	1007.9	373.485	1.417	
9	2-Pentyl-furan	C3777693	C_9_H_14_O	138.2	991.9	354.060	1.262	
10	Trimethylpyrazine	C14667551	C_7_H_10_N_2_	122.2	1012.2	379.259	1.163	
11	1-Octen-3-ol	C3391864	C_8_H_16_O	128.2	983.9	346.185	1.166	*M*
12	Benzaldehyde	C100527	C_7_H_6_O	106.1	965.3	328.335	1.153	*M*
13	Octanal	C124130	C_8_H_16_O	128.2	1005.5	370.335	1.841	
14	Alpha-phellandrene	C99832	C_10_H_16_	136.2	988.2	350.384	1.699	
15	(E)-2-Heptenal	C18829555	C_7_H_12_O	112.2	961.4	324.660	1.682	
16	1-Octen-3-ol	C3391864	C_8_H_16_O	128.2	983.4	345.660	1.617	*D*
17	Benzaldehyde	C100527	C_7_H_6_O	106.1	961.9	325.185	1.482	*D*
18	2-Heptanone	C110430	C_7_H_14_O	114.2	886.2	262.815	1.272	*M*
19	Heptanal	C111717	C_7_H_14_O	114.2	905.5	276.990	1.339	
20	2-Heptanol	C543497	C_7_H_16_O	116.2	897.3	270.585	1.708	
21	2-Heptanone	C110430	C_7_H_14_O	114.2	889.4	264.914	1.642	*D*
22	2-Methylpyrazine	C109080	C_5_H_6_N_2_	94.1	829.9	229.005	1.089	
23	3-Hexen-1-ol	C928961	C_6_H_12_O	100.2	854.3	243.075	1.511	
24	2-Hexanol	C626937	C_6_H_14_O	102.2	793.3	209.370	1.576	
25	2,3-Butanediol	C513859	C_4_H_10_O_2_	90.1	790.2	207.795	1.373	
26	2-Butanone	C78933	C_4_H_8_O	72.1	577.3	131.040	1.254	
27	Butanal	C123728	C_4_H_8_O	72.1	601	137.340	1.299	
28	Ethyl acetate	C141786	C_4_H_8_O_2_	88.1	606.7	138.915	1.349	
29	2-Methylbutanal	C96173	C_5_H_10_O	86.1	649.3	151.200	1.406	
30	1,4-Dioxane	C123911	C_4_H_8_O_2_	88.1	701.5	168.525	1.328	
31	1,2-Dimethoxyethane	C110714	C_4_H_10_O_2_	90.1	647.2	150.569	1.314	
32	Ethanol	C64175	C_2_H_6_O	46.1	453.1	102.375	1.131	
33	2-Octanol	C123966	C_8_H_18_O	130.2	986	348.180	1.439	
34	(E)-2-Hexenal	C6728263	C_6_H_10_O	98.1	830.1	229.109	1.187	
35	Butanoic acid	C107926	C_4_H_8_O_2_	88.1	809.9	218.085	1.156	
36	2-Methylpropanoic acid	C79312	C_4_H_8_O_2_	88.1	763	194.775	1.159	
37	Decanal	C112312	C_10_H_20_O	156.3	1206.8	754.529	1.54	
38	2-Phenylethanol	C60128	C_8_H_10_O	122.2	1109.2	534.450	1.523	
39	2-Phenylacetaldehyde	C122781	C_8_H_8_O	120.2	1043.2	423.150	1.25	
40	Heptanol	C53535334	C_7_H_16_O	116.2	979	341.355	1.388	
41	Isoamyl acetate	C123922	C_7_H_14_O_2_	130.2	889.3	264.810	1.756	
42	2-Methylbutanoic acid	C116530	C_5_H_10_O_2_	102.1	846.3	238.350	1.467	
43	Pentanoic acid	C109524	C_5_H_10_O_2_	102.1	915.2	284.759	1.224	
44	Thiazole	C288471	C_3_H_3_NS	85.1	744.5	186.480	1.261	
45	2-Methylpropanol	C78831	C_4_H_10_O	74.1	624.3	143.849	1.171	
46	2,3-Butanedione	C431038	C_4_H_6_O_2_	86.1	564.3	127.679	1.161	
47	Linalool	C78706	C_10_H_18_O	154.3	1102.9	522.690	1.226	
48	Styrene	C100425	C_8_H_8_	104.2	889.9	265.229	1.514	
49	n-Nonanal	C124196	C_9_H_18_O	142.2	1100.8	518.700	1.483	*M*
50	Cyclohexanone	C108941	C_6_H_10_O	98.1	887.7	263.759	1.462	
51	2-Ethyl pyrazine	C13925003	C_6_H_8_N_2_	108.1	913	282.975	1.52	
52	Ethyl propionate	C105373	C_5_H_10_O_2_	102.1	693.5	165.375	1.442	
53	(Z)-3-Hexenyl acetate	C3681718	C_8_H_14_O_2_	142.2	1002.3	366.240	1.802	
54	Ethyl 2-hydroxypropanoate	C97643	C_5_H_10_O_3_	118.1	810.5	218.400	1.541	
55	Butyl acetate	C123864	C_6_H_12_O_2_	116.2	795.3	210.420	1.62	

*Note.* RI is the retention index, Rt is the retention time, Dt is the migration time, and (RIP rel) is the normalization treatment. *D* is a dimer, and *M* is a monomer. The sequence numbers of components in Figures4–7 are the same as those in the table.

## Data Availability

The odor composition data tables used to support the results of this study are included in this article.

## Abbreviations

GGEC: Galli gigeriae endothelium corneum
SP: Raw galli gigerii endothelium corneum
QC: Stir-fried galli gigeriae endothelium corneum
ST: Sand ironing galli gigeriae endothelium corneum
CZ: Stir-fried galli gigeriae endothelium corneum with vinegar
FH: Stir-fried galli gigeriae endothelium corneum with milk
PCA: Principal component analysis
OPLS-DA: Orthogonal partial least squares discriminant analysis
VOCs: Volatile organic compounds
CAS: Chemical abstracts service.
